# Impact of COVID-19 on the cost of surgical and obstetric care: experience from a Nigerian teaching hospital and a review of the Nigerian situation

**DOI:** 10.11604/pamj.supp.2020.37.1.25935

**Published:** 2020-09-22

**Authors:** AbdulHafiz Oladapo Adesunkanmi, Akaninyene Eseme Ubom, Olalekan Olasehinde, Olusola Benjamin Fasubaa, Omotade Adebimpe Ijarotimi, Abdul Rashid Kayode Adesunkanmi, Nwedobong Ededem Okon

**Affiliations:** 1 Department of Surgery, Obafemi Awolowo University Teaching Hospitals Complex, Ile-Ife, Osun State, Nigeria,; 2 Department of Obstetrics, Gynaecology, and Perinatology, Obafemi Awolowo University Teaching Hospitals Complex, Ile-Ife, Osun State, Nigeria,; 3 Department of Surgery, Obafemi Awolowo University, Ile-Ife, Osun State, Nigeria,; 4 Department of Obstetrics, Gynaecology, and Perinatology, Faculty of Clinical Sciences, College of Health Sciences, Obafemi Awolowo University, Ile-Ife, Osun State, Nigeria,; 5 Medstar Medical Group, World Bank, Washington DC, USA

**Keywords:** Impact, cost, COVID-19, surgical and obstetric care, Nigerian teaching hospital

## Abstract

The public health impact of the COVID-19 pandemic cannot be overstated. Its impact on the cost of surgical and obstetric care is significant. More so, in a country like Nigeria, where even before the pandemic, out-of-pocket spending (OOPS) has been the major payment method for healthcare. The increased cost of surgical and obstetric care occasioned by the pandemic has principally been due to the additional burden of ensuring the use of adequate/appropriate personal protective equipment (PPE) during patient care as a disease containment measure. These PPE are not readily available in public hospitals across Nigeria. Patients are therefore compelled to bear the financial burden of procuring scarce PPE for use by health care personnel, further increasing the already high cost of healthcare. In this study, we sought to appraise the impact of the COVID-19 pandemic on the cost of surgical and obstetric care in Nigeria, drawing from the experience from one of the major Nigerian teaching hospitals- the Obafemi Awolowo University Teaching Hospitals Complex (OAUTHC), Ile-Ife, Osun State. The cost of surgical and obstetric care was reviewed and compared pre- and during the COVID-19 pandemic, deriving relevant examples from some commonly performed surgical operations in our centre (OAUTHC). We reviewed patients' hospital bills and receipts of consumables procured for surgery. Our findings revealed that the cost of surgical and obstetric care during the COVID-19 pandemic had significantly increased. We identified gaps and made relevant recommendations on measures to reduce the additional costs of surgical and obstetric care during and beyond pandemic.

## Essay

In late December 2019, the emergence of a novel coronavirus disease-2019 (COVID-19), previously named 2019 novel coronavirus (2019-nCoV), was reported in Wuhan, Hubei province, China [1]. By January 30, 2020, when the World Health Organisation (WHO) announced COVID-19 as the sixth Public Health Emergency of International Concern, the disease had spread to over 20 countries of the world, including the United States of America (USA) and Europe [1]. It was declared a pandemic on March 11, 2020, having fulfilled the epidemiological criteria of more than 100,000 infections in at least 100 countries [2]. Currently, over 23 million cases and more than 800,000 deaths have been recorded in 215 countries of the world [3]. Every African country is currently affected by the disease, with more than 1 million cases and over 27,000 deaths so far reported in Africa, representing about 5% of all cases recorded globally [3]. Nigeria reported her first confirmed case of COVID-19, the first in sub-Saharan Africa, on February 27, 2020 [4]. Since then, there has been an upward trajectory in the rate of infection across the country owing to widespread community transmission [5]. As of August 29, 2020, 53,727 cases and 1,011 deaths had been recorded in all 36 States of Nigeria, including the Federal Capital Territory (FCT) [6]. Before the COVID 19 pandemic, the Nigerian public health care system had been grossly under-equipped and underfunded with major implications on health care indices such as mortalities from obstetric, surgical, trauma-related, or other potentially curable health conditions. The additional strain put on the already fatigued system by the pandemic certainly has the potential of having major implications on access to quality health care [7]. Given the very limited coverage of the National Health Insurance Scheme (NHIS), which necessitates out-of-pocket spending (OOPS) even in emergencies [8], the odds are certainly against better outcomes. This study sought to appraise the impact of COVID-19 on the cost of surgical and obstetric care in Nigeria, drawing from the experience from one of the major Nigerian teaching hospitals- the Obafemi Awolowo University Teaching Hospitals Complex (OAUTHC), Ile-Ife, Osun State.

**Healthcare financing and funding in Nigeria:** Universal Health Coverage (defined by the WHO as “access to key promotive, preventive, curative, and rehabilitative health interventions for all at an affordable cost, thereby achieving equity in access” [9]) service coverage index in Nigeria is currently estimated at 39% [10]. Nigeria´s foremost Universal Health Coverage scheme, the National Health Insurance Scheme (NHIS), though signed into law in 1999, only became fully operational six years later, in 2005. Enrolment in the scheme since its inception to date has remained suboptimal, as only about 5 million Nigerians (less than 3% of the population) are currently enrolled [10]. This number represents mostly those in formal employment, who are mandatorily enrolled in the scheme. Many Nigerians are however either unemployed or work in the non-formal sector [11]. The grimmer reality is that about 70% of Nigerians live on less than $1 a day, with approximately 35% of the 70% living in absolute poverty, while over 90% live on less than $2 a day [10]. Nigerian workers currently earn a paltry ₦30,000 ($79) as minimum wage [12]. The mostly poor patients still must pay for healthcare from their already depleted pockets. Worse still, asides out-of-pocket payments for healthcare services rendered in our public hospitals, patients are still made to buy the most basic of medical consumables [13]. Health was allocated only a meagre 4.14% of Nigeria's 2020 budget, a far cry from the recommended benchmark of 15% [14].

### Increasing cost of surgical and obstetric care occasioned by the COVID-19 pandemic

**Surgical care:** sequel to the lockdown imposed by the Nigerian government on March 30, 2020 (which is currently being eased), as well as other disease containment measures, elective operations in many public hospitals across the country were postponed/cancelled [15,16]. This is in line with recommendations of the American College of Surgeons and the Royal College of Surgeons of England, to postpone or delay non-emergency procedures except cancer-related operations [17,18]. It must however be borne in mind that delaying some elective cases may advance the pathology or result in complications requiring emergency interventions with increased costs and likelihood of poorer outcomes [15,16]. The postponement of elective surgeries saw a sharp drop in surgical procedures in public hospitals across the country [16]. In our centre, only 146 elective surgical (cardiothoracic- 28, paediatric surgical- 29, urologic- 15, and general surgery- 74) procedures were undertaken between March 2020 and July 2020. This is a third of the number of procedures done within the same timeframe in 2019. The declining numbers notwithstanding, healthcare costs have remained high (and increasing) owing to the additional costs of protective equipment including N95 face masks, which are in short supply [19]. The rising financial burden of surgical and obstetric care in the country is accentuated by widespread poverty, abysmal government funding of public healthcare, and suboptimal health insurance as already highlighted [7,8,10,12].

In our centre, patients scheduled for elective surgeries are now mandatorily required to be screened for COVID-19, in line with testing recommendations for patients planned for surgery [20,21]. The reverse-transcription polymerase chain reaction (RT-PCR) test, which is the gold standard [20], if done in the Nigeria Centre for Disease Control (NCDC) accredited non-fee paying testing centres is free [22]. These centres are however grossly inadequate, as there are currently only 63 NCDC accredited non-fee paying COVID-19 testing laboratories in 32 out of 36 States of the country (including the FCT) [23], serving a total population of over 200 million. Delays in testing and release of results are therefore not uncommon, owing to inadequate testing facilities [24]. More so, testing in the country is not universal [24] but largely restricted to suspected cases of COVID-19, defined by the WHO as “a patient with acute respiratory illness (fever and at least one sign/symptom of respiratory disease e.g. cough, shortness of breath), AND with no other etiology that fully explains the clinical presentation AND a history of travel to or residence in a country/area or territory reporting local transmission of COVID-19 disease during the 14 days prior to symptom; OR A patient with any acute respiratory illness AND having been in contact with a confirmed or probable COVID-19 case in the last 14 days prior to onset of symptoms; OR A patient with severe acute respiratory infection ((fever and at least one sign/symptom of respiratory disease (e.g. cough, shortness of breath) AND requiring hospitalization AND with no other etiology that fully explains the clinical presentation” [25]. Due to the paucity of testing kits and facilities, even suspected cases are often times triaged for testing based on symptoms and epidemiological risk, as per the triage algorithm proposed for low-income settings with limited testing capacity [26].

As of August 13, 2020, only 341,421 tests had been done in Nigeria [6], representing less than 0.25% of her total population of over 200 million. Countries like Ghana and South Africa, with far lesser populations, have completed more tests [7,27]. Not all patients can therefore be tested for free in the NCDC accredited non-fee paying testing laboratories prior to surgery. Patients who can afford the cost are therefore compelled to do these antigen tests in privately-owned testing laboratories, where the cost is as high as ₦50,400 ($132) [22]. To make matters worse, some of these laboratories charge extra fees of between ₦10,000 and ₦50,000 for so-called “logistics”, “home service” or “mobilization” [22]. The implication of this is that clients stand to pay as much as between ₦60,400 ($159) and ₦100,400 ($264) if these extra charges are factored in [22]. This is two to three times the monthly minimum wage, four to six times the cost of an excisional biopsy (₦17,000, $44.7), three to four times the cost of a herniorrhaphy (₦23,000, $60.4), and almost twice the cost of an appendectomy (₦48,500-₦55,650, $127.5-$146.3) in our centre. For very indigent patients who cannot be tested for free in an NCDC-accredited laboratory, and who also cannot afford the cost of RT-PCR in private laboratories, COVID-19 antibody tests are accepted in our centre for selected cases perceived to be low risk. Antibody tests are also done for emergency surgeries. The antibody test, though not as clinically useful as the antigen test, is more readily available, cheaper, and results are available in as little as 15 minutes [20]. The antibody test is done in our centre for ₦5,000 ($13).

Patients for surgery, in addition, have to buy/pay for most of the consumables/materials needed for surgery, which include but are not limited to gloves, surgical gowns, goggles, and face masks [28]. Even patients on NHIS still suffer catastrophic health expenditure (CHE), defined as spending on healthcare beyond a certain income threshold, as they most times still have to purchase these additional materials out of their pockets, either because they are out of stock in the hospital pharmacies or they are not covered under the NHIS [10,28]. In our centre, before the COVID-19 pandemic, patients were required to buy three surgical gowns, one each for the surgeon, his assistant, and the scrub nurse. The circulating nurse, the anesthetist, the anesthetist assistant, and the theatre attendant used sterile single-use aprons. Regular surgical masks, which are relatively cheap, were hitherto used for surgeries. Currently, however, as part of the COVID-19 infection control protocol, all our operating room staff mandatorily wear surgical gowns and at least KN95 face masks (especially so for emergency surgeries, when RT-PCR testing is not feasible), in line with Personal Protective Equipment (PPE) requirement for surgical teams, which should include a fitted N95 respirator mask and droplet PPE (gown, gloves, eye protection) [21]. Patients are therefore now required to buy seven surgical gowns (four more than the previous three), one each for the surgeon and his assistant, the scrub and circulating nurses, the anesthetist and his assistant, as well as the theatre attendant. Each surgical gown is sold for between ₦3,500-₦4,000 ($9-$10.5) [29]. Patients therefore spend an additional ₦15,000 ($39.4) on surgical gowns on average. This amount is one-half the country's minimum wage [12]. Additionally, patients now also have to buy nine KN-95 facemasks (three each for the surgeons, nurses, and anesthetists, inclusive of the theatre attendants), sold at a unit price of about ₦850 ($2.2) [30]. Nine of these masks cost about ₦7,650 ($20.1). Similarly, in the Lagos University Teaching Hospital (LUTH), Lagos, Nigeria, patients spend as much as ₦74,400 ($196) buying additional consumables for surgery, including nine N95 masks, nine disposable surgical gowns, and four disposable goggles [28].

Where a patient requires a radiological/ sonographic examination prior to surgery, PPE, consisting of a surgical gown, face mask, and a pair of surgical gloves, is also purchased for the use of the radiologist/sonologist in our centre. These together cost about ₦5,000 ($13). An abdominopelvic ultrasound scan costs ₦3,500 ($9.2) while a soft tissue ultrasound scan is done for ₦4,000 ($10.5) in our centre. “The cost of procuring PPE for the radiologist/sonologist is therefore more than the cost of these ultrasonographic investigations”. Even though the Nigerian government has recently made significant efforts in increasing the supply of PPE to public hospitals across the country, these are still grossly inadequate [7]. The burden of purchasing scarce PPE is therefore passed down to the poor and hapless patients [28]. A patient who during this pandemic is booked for a herniorrhaphy in our centre for instance, may additionally spend up to ₦128,050 ($337), that is, up to ₦100,400 ($264) for the now mandatory preoperative COVID-19 test (if done in a private laboratory), ₦15,000 ($39.4) for four extra surgical gowns and ₦7,650 ($20.1) for nine KN-95 face masks (for the operating room personnel), as well as ₦5,000 ($13) for PPE for the sonologist for an abdominopelvic ultrasound scan if required preoperatively. This additional cost is nearly six times the cost of a herniorrhaphy, which is currently ₦23,000 ($60.4) (Table 1).

**Table 1:** cost of some commonly performed surgeries and medical consumables for surgeries in our centre pre- and during the COVID-19 pandemic

	Appendectomy		Herniorrhaphy		Excisional biopsy		Caesarean section	
	Pre-COVID- 19, ₦ ($)	COVID-19, ₦ ($)	Pre- COVID-19, ₦ ($)	COVID-19, ₦ ($)	Pre- COVID-19, ₦ ($)	COVID-19, ₦ ($)	Pre-COVID-19, ₦ ($)	COVID-19, ₦ ($)
Cost of surgery (inclusive of anesthesia and surgical pack)	48,500-55,650 (127.5-146.3)	48,500-55,650 (127.5-146.3)	23,000 (60.4)	23,000 (60.4)	17,000 (44.7)	17,000 (44.7)	30,000-35,000 (78.8-92.0)	30,000-35,000 (78.8-92.0)
Surgical gowns (at a unit price of ₦3,500 - ₦4,000)	x 3 = 10,500-12,000 (27.6-31.5)	x 7 = 24,500-28,000 (64.4-73.6)	x 3 = 10,500-12,000 (27.6-31.5)	x 7 = 24,500-28,000 (64.4-73.6)	x 3 =10, 500-12, 000 (27.6-31.5)	x 7 = 24,500-28,000 (64.4-73.6)	x 3 = 10,500-12,000 (27.6-31.5)	x 7 = 24,500-28,000 (64.4-73.6)
Regular surgical masks (at a unit price of about ₦1,000/pack)	1,000 (2.6)	-	1,000 (2.6)	-	1,000 (2.6)	-	1,000 (2.6)	-
KN 95 masks (at a unit price of ₦850)	-	x 9 = 7,650 (20.1)	-	x 9 = 7,650 (20.1)	-	x 9 = 7,650 (20.1)	-	x 8 = 6,800 (17.9)
PPE for radiologic investigation/obstetric scan (if required)	-	5,000 (13.1)	-	5,000 (13.1)	-	5,000 (13.1)	-	5,000 (13.1)
COVID-19 testing (RT-PCR) before surgery	-	up to 100,400 (263.9)	-	up to 100,400 (263.9)	-	up to 100,400 (263.9)	-	-
Pre-admission COVID-19 antibody testing	-	-	-	-	-	-	-	5,000 (13.1)
**Total**	60,000-68,650 (157.7-180.4)	186,050-196,700 (489-517)	34,500-36,000 (90.7-94.6)	160,550-164,050 (421.9-431.1)	28,500-30,000 (74.9-78.8)	154,550-158,050 (406.2-415.4)	41,500-48,000 (109.1-126.1)	71,300-79,800 (187.4-209.7)

**Obstetric care:** in our centre, COVID-19 screening is currently not a prerequisite for elective Caesarean section, though this is presently being considered. Patients scheduled for a Caesarean section currently bear an additional cost of about ₦15,000 ($39.4) for four extra surgical gowns (one each for the anesthetist and his assistant, circulating nurse, and attending neonatologist, all of whom hitherto used sterile single-use aprons), ₦6,800 ($17.9) for eight KN-95 face masks (one each for the surgeon, surgeon´s assistant, anesthetist, anesthetist assistant, scrub nurse, circulating nurse, attending neonatologist and theatre attendant) and ₦5,000 for PPE for the sonologist for an obstetric ultrasound (often requested preoperatively to confirm foetal lie and presentation, foetal biometrics/estimated foetal weight, location of the placenta, and lower uterine segment thickness in case of a previous Caesarean section, amongst other indications). This brings the total additional cost to about ₦26,800 ($70), which is almost the cost of a Caesarean section in our centre. A Caesarean section in our centre costs between ₦30,000-₦35,000 ($79-$92). The additional cost of procuring PPE for use by the sonologist for sonographic examinations is multiplied by the number of ultrasound scans an obstetric patient would require in the antenatal period. On average, we request three obstetric scans in pregnancy. An early scan, usually between 11^+0^-13^+6^ weeks to date pregnancy, ascertain foetal viability and confirm chorionicity in case of multiple pregnancy; a foetal anatomy scan between 18 and 20 weeks; and a third trimester ultrasound scan. Additional scans may be requested for other obstetric reasons and in high risk pregnancies. For every of these scans, the patient spends an additional ₦5,000 ($13) on PPE for the sonologist. This is almost twice the cost of an obstetric scan in our centre, which is currently ₦2,550 ($6.7). Due to the increasing spate of patients developing COVID-19 symptoms while on admission for other obstetric reasons, and subsequently testing positive for COVID-19, it is now mandatory in our obstetric unit, that all obstetric patients who require admission do a COVID-19 antibody test. This translates to an additional cost of ₦5,000 ($13), borne by the patient, which is the cost of the COVID-19 antibody test in our centre (Table 1).

## Conclusion

The COVID-19 pandemic has exponentially escalated the cost of surgical and obstetric care in Nigeria. This is necessitated by the additional cost of COVID-19 infection control measures/protocols, including mandatory preoperative COVID-19 testing, use of surgical gowns and KN-95 face masks by all operating room personnel, as well as the use of PPE kit by radiologists/sonologists. These additional costs are compulsorily passed to the mostly indigent and hapless patients because poor funding of public healthcare by the government mitigates against the inclusion in surgical packs/availability of medical consumables for surgery (including surgical gowns and face masks) in public hospitals in the country. Furthermore, enrolment in the NHIS is poor. Only approximately 3% of Nigerians are currently enrolled in the scheme. There is also widespread poverty in the country, as up to 70% of Nigerians live on less than a dollar per day, with a minimum wage of ₦30,000 ($79).

**Recommendations:** there is a need for public economic policies that would engender economic growth and development and make more jobs available for the teeming unemployed populace. There is also a need for the government to increase the minimum wage from the current meagre ₦30,000 ($79). These measures would put more money in the pockets of patients to pay for healthcare. Steps should be taken to strengthen and upscale enrolment in the NHIS. Nigerians should be made to understand the scheme better, to improve uptake. Payment policies may have to be adapted to favour enrolment by the bulk of Nigerians who work in the non-formal/private sectors. Health-related savings should be encouraged amongst non-enrollees. It is recommended that the government invests more in health, beginning with ensuring allocation of the recommended 15% of the total budget to health. A legislative back-up to make this mandatory may become necessary. Private investors should also be encouraged to invest in public health care services. The government should increase the supply of PPE to public hospitals across the country. It should also provide incentives to indigenous manufacturers to produce PPE in line with recommended standards, from local materials and technology. This would go a long way in reducing the cost of these PPE, as well as the overall cost of surgical and obstetric care, and provide more jobs to boost the economy. There is a need to increase our COVID-19 testing capacity and decentralize tests to improve access and reduce delays in testing and release of test results. The cost of tests in accredited fee-paying laboratories should be regulated and affordable. There is no gainsaying the fact that the COVID-19 pandemic has further strained the grossly underfunded and under-equipped public healthcare system in Nigeria. A complete overhaul and revamp of existing infrastructure and services is the panacea at this point in time.
